# Treatment of complex limb fractures with 3D printing technology combined with personalized plates: a retrospective study of case series and literature review

**DOI:** 10.3389/fsurg.2024.1383401

**Published:** 2024-05-16

**Authors:** Hairui Liang, Beibei Chen, Siyu Duan, Lei Yang, Rongda Xu, He Zhang, Ming Sun, Xueting Zhou, Hanfei Liu, Hang Wen, Zhencun Cai

**Affiliations:** ^1^ Department of Orthopedics Surgery, Central Hospital Affiliated to Shenyang Medical College, Shenyang, Liaoning, China; ^2^ School of Pharmacy, Inner Mongolia Medical University, Inner Mongolia Autonomous Region, Shenyang, China; ^3^ Key Laboratory of Human Ethnic Specificity and Phenomics of Critical Illness in Liaoning Province, Shenyang Medical College, Shenyang, China

**Keywords:** 3D printing technology, personalized custom steel plates, limb fractures, preoperative planning, case series, literature review

## Abstract

**Background:**

In recent years, 3D printing technology has made significant strides in the medical field. With the advancement of orthopedics, there is an increasing pursuit of high surgical quality and optimal functional recovery. 3D printing enables the creation of precise physical models of fractures, and customized personalized steel plates can better realign and more comprehensively and securely fix fractures. These technologies improve preoperative diagnosis, simulation, and planning for complex limb fractures, providing patients with better treatment options.

**Patients and methods:**

Five typical cases were selected from a pool of numerous patients treated with 3D printing technology combined with personalized custom steel plates at our hospital. These cases were chosen to demonstrate the entire process of printing 3D models and customizing individualized steel plates, including details of the patients' surgeries and treatment procedures. Literature reviews were conducted, with a focus on highlighting the application of 3D printing technology combined with personalized custom steel plates in the treatment of complex limb fractures.

**Results:**

3D printing technology can produce accurate physical models of fractures, and personalized custom plates can achieve better fracture realignment and more comprehensive and robust fixation. These technologies provide patients with better treatment options.

**Conclusion:**

The use of 3D printing models and personalized custom steel plates can improve preoperative diagnosis, simulation, and planning for complex limb fractures, realizing personalized medicine. This approach helps reduce surgical time, minimize trauma, enhance treatment outcomes, and improve patient functional recovery.

## 1 Introduction

Complex limb fractures refer to fractures with intricate patterns, which may involve multiple fracture sites, severe dislocations, comminuted fractures, or joint surface involvement. Complex limb fractures are relatively common due to the diverse causes of fractures and the substantial mechanical stresses that limbs endure during physical activities. Consequently, they represent a significant proportion of all fracture cases, with a substantial number of patients affected (1, 2). In the case of limb fractures, the severity of the fracture and the complex anatomical structures involved can make the treatment of some limb fractures quite challenging (3). In recent years, 3D printing technology has matured, allowing orthopedic surgeons to use preoperative CT data to print visual models of fractures, thereby enabling more comprehensive preoperative diagnosis, simulation, and surgical planning (4–6). For some complex limb fractures, 3D printing technology can be used to design personalised implants, such as custom plates, to achieve individualized and precise treatment. This helps in reducing surgical time, improving surgical efficacy, lowering trauma, reducing postoperative complications, and facilitating postoperative functional recovery (7–9).

The diagnosis and treatment of limb fractures depend on several factors, including the type and location of the fracture, the degree of displacement, the patient's age, and overall health. Common treatment methods include external support devices (such as casts), external fixation devices, internal fixation (such as plates, screws, intramedullary nails, Kirschner wires, wires, etc.), and surgical interventions in complex cases (involving open fractures or articular surface involvement) (10–15). In recent years, the field of orthopedics has rapidly advanced, and with the further dissemination of fracture classification and treatment concepts, limb fracture diagnosis and treatment have established a relatively comprehensive system. This system allows for the selection of the most suitable treatment approach for patients based on fracture classification and type (16, 17). However, some complex limb fractures, characterized not only by the severity of the fractures but also by unfavorable soft tissue conditions, continue to present significant challenges for conventional treatment methods. The selection and customisation of the optimal treatment approach should be based on the individual patient's specific circumstances and the characteristics of the fracture (18, 19).

3D printing technology is an advanced manufacturing technique widely used in various fields. It is based on the principle of additive manufacturing, where digital design files are transformed into physical objects by layer-by-layer stacking or incremental addition of materials (20). In the field of orthopedics, the manufacturing process involves image acquisition, image post-processing, and 3D printing (21). There are various 3D printing technologies, including Selective Laser Sintering (SLS), Stereolithography (SLA), Fused Deposition Modeling (FDM), Selective Deposition Modeling (SDM), Bioprinting, Electron Beam Melting (EBM), and Direct Metal Laser Sintering (DMLS). Different printing technologies have their respective advantages, and the appropriate technology can be selected based on specific requirements (22–27). 3D printing technology has had a revolutionary impact on the field of orthopedics, providing a wide range of applications, including preoperative planning, customized implants, surgical simulations, fracture modeling, bioprinting, surgical navigation, and more. It has also equipped healthcare professionals with additional tools and resources and transforming the methods of orthopedic medical care and surgery. This technology has introduced numerous innovative and personalised solutions to enhance patient treatment and recovery processes (6, 7, 27–35). Despite the numerous advantages of 3D printing, it also faces certain challenges and limitations. 3D printing technology is continually evolving, and currently, there is ongoing exploration of new materials, faster printing methods, and broader application areas (36). This technology holds the promise of further enhancing the quality and efficiency of orthopedic healthcare in the future.

## 2 Patients and methods

Five representative cases were selected from a pool of numerous patients undergoing treatment with 3D printing technology combined with personalized custom steel plates at our institution. (3D Group) These cases were chosen to comprehensively demonstrate the entire process of printing 3D models and customizing individualized steel plates, including an overview of the patients' surgeries, treatment procedures, and postoperative follow-up outcomes.

This study was conducted with the approval of the Ethics Committee of the Affiliated Central Hospital of Shenyang Medical College. Patients provided informed consent for the publication of case details and any accompanying images, in accordance with publication terms.

### 2.1 The process of customizing individualized steel plates

The patient's fracture CT data was imported into Mimics 20.0 software (Materialise, Belgium) workstation in DICOM format for three-dimensional modeling. The fracture fragments were separated and color-coded using the thresholding selection function in the software, and then anatomical virtual reduction of the fracture fragments was performed using the move and rotate functions. Subsequently, the three-dimensional modeling and virtually reduced fracture data were exported as STL files and imported into FlashPrint 5 software (Flashforge, China) to print physical models using PLA (polylactic acid) as the printing material.

Through collaboration between biomedical engineers and surgeons, based on the virtually reduced fracture model, customized individualized steel plate solutions were developed using Unigraphics NX (Siemens PLM Software, USA). The optimal placement of the steel plates was determined to achieve sufficient reduction and strong fixation of the fracture area. Specific steel plate design parameters included the fixation area, shape, curvature, thickness, and the positions and orientations of screw holes.

Next, the physical models of the steel plates were printed using PLA as the printing material with FlashPrint 5 software. The physical models of the steel plates were then reverse-scanned and imported into Mimics software. The placement and fixation of the steel plates were simulated on the virtually reduced three-dimensional model, confirming the positions, lengths, directions, and diameters of the screws. Transparency processing was applied to the fracture model to ensure proper screw length and positioning without intruding into the joint cavity. The length of the fixation screws was measured using the measurement function, and the recommended screw lengths were marked next to each screw hole on the steel plate.

Based on the designed individualized steel plate production drawings, the steel plates were manufactured using pure titanium TA3 as the raw material. After fabrication, the steel plates underwent processes such as sandblasting, magnetic polishing, and ultrasonic cleaning before being sent to a specialized quality inspection department for testing. After passing quality inspection, the individualized steel plates were labeled and packaged. Prior to surgery, the steel plates underwent high-temperature and high-pressure sterilization for disinfection and were kept on standby during the procedure.

### 2.2 Surgical technique

All patients underwent surgery performed by the same group of surgeons. Preoperative vital signs, postoperative imaging, and functional assessments during follow-up were conducted collectively by three physicians from our team. The only difference compared to previous treatments for limb fractures was the use of 3D printing technology in conjunction with individualized customized steel plates; no other interventions were applied. Detailed surgical procedures are described in each case presentation.

### 2.3 Baseline data and follow-up assessment indicators

A paired case-control analysis was conducted, matching 3D group cases with non-3D group patients based on fracture type, age, gender, and BMI. The shortest follow-up period for all participants in the study was one year. Postoperative x-rays were reviewed on the first day after surgery to assess fracture reduction quality. To compare the surgical time, fracture healing time, postoperative three-month joint function score, and complication rates between the two groups of paired cases, as well as to assess the postoperative functional recovery, joint function scores, and activities of daily living (ADL) at one year postoperatively.

Functional scores were assessed according to the Mayo score for the elbow joint (37), the Cooney score for the wrist joint (38), the Hospital for Special Surgery (HSS) score for the knee joint (39), the Mazur score for the ankle joint (40), and the postoperative modified Neer score for the shoulder joint (41).

Fracture reduction quality was classified as excellent (angle less than 5° or displacement less than 2 millimeters in any plane), good (angle between 5° and 10° or displacement between 2 and 5 mm in any plane), fair (angle between 10° and 20° or displacement between 5 and 10 mm in any plane), and poor (angle greater than 20° or displacement greater than 10 mim) (42).

### 2.4 Statistical analysis

Statistical analysis was conducted using SPSS version 27.0 software. Descriptive statistics included the mean and standard deviation for continuous variables, as well as frequencies for categorical variables. The Shapiro-Wilk test was employed to assess normality. For categorical variables, the chi-square test was used for analysis, while for continuous variables, both independent samples *t*-test and Mann-Whitney *U*-test were utilized. The significance level was set at *p* < 0.05.

## 3 Results

Figures 1–10 illustrate the schemes of 3D-printed fracture models and customized personalized steel plates.

**Figure 1:**
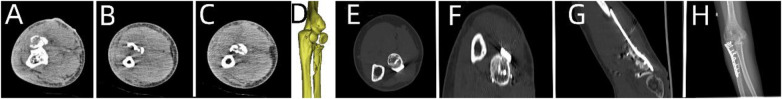
Preoperative and postoperative radiological data of the patient. (**A–D**) Preoperative CT images of the patient. (**E–G**) CT images at 7 months postoperative. (**H**) x-ray at 7 months postoperative.

**Figure 2:**
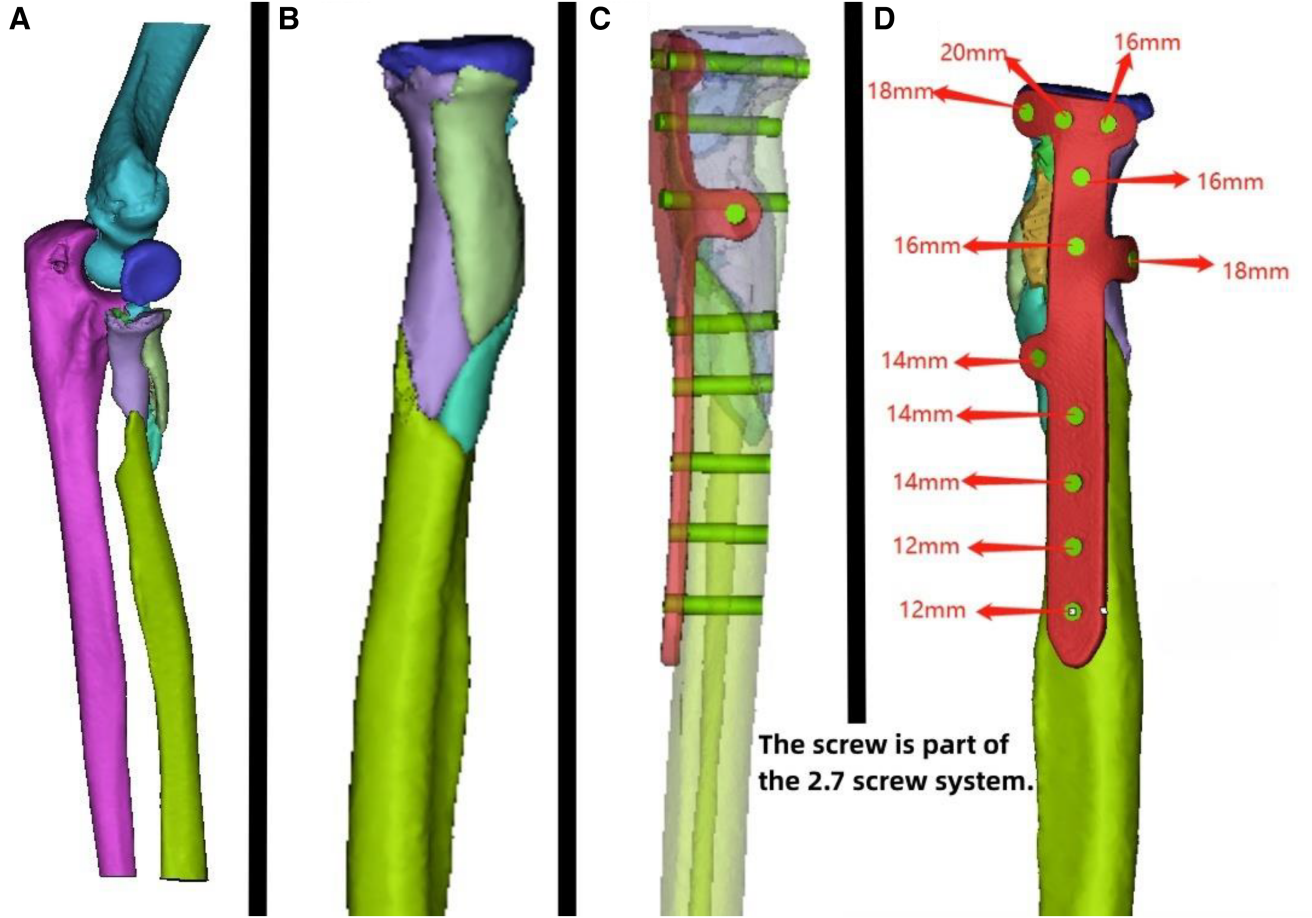
Patient's 3D printing and custom plate data. (**A**) Virtual simulation of preoperative fracture CT data. (**B**) Virtual reduction of patient's fracture data. (**C,D**) Design of the plate and screws for the patient, simulation of plate positioning, and data for screw length measurement.

**Figure 3:**
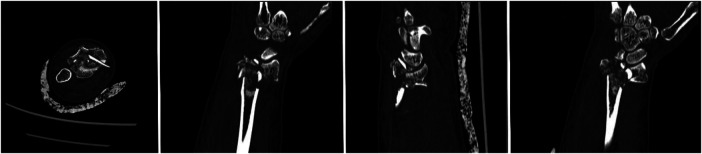
Patient's preoperative CT scan of the fracture.

**Figure 4:**
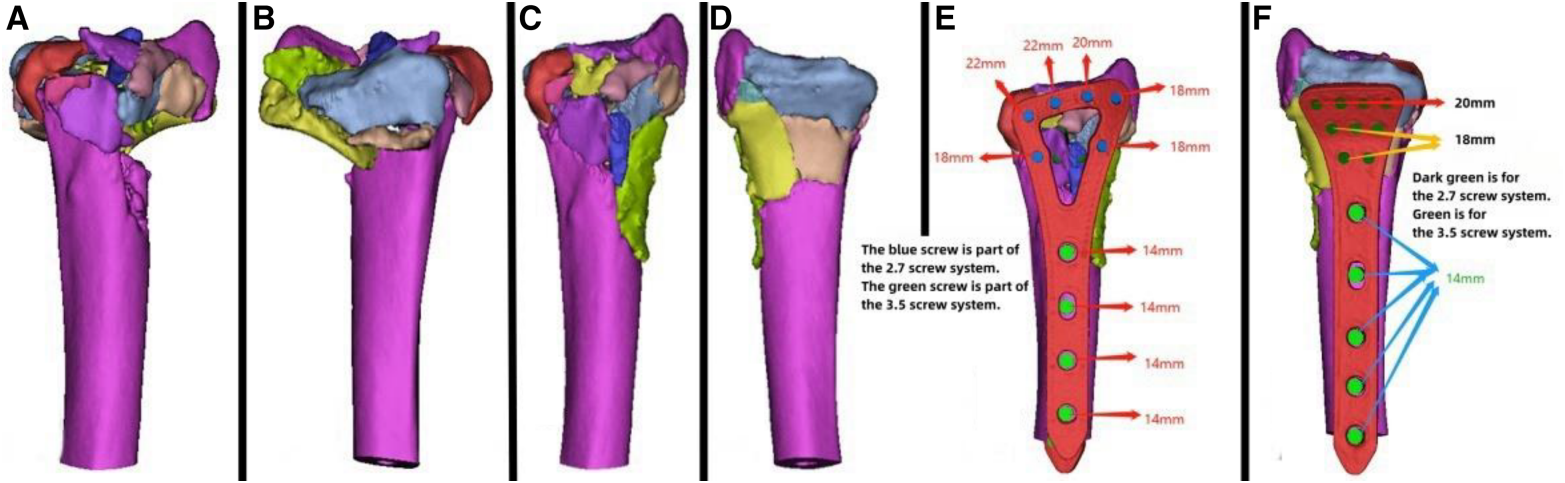
Patient's 3D printing and customized steel plate data. (**A,B**) Virtual simulation of preoperative CT data. (**C,D**) Data of fracture reduction in the patient. (**E,F**) Design of the steel plate and screws, simulation of the steel plate's position, and data for screw length measurement.

**Figure 5:**
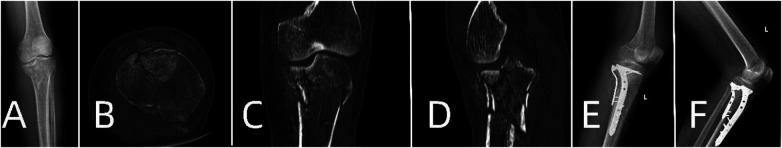
Patient's preoperative and postoperative radiological data. (**A**) Preoperative x-ray. (**B–D**) Preoperative CT scans. (**E**) x-ray on the first day postoperatively. (**F**) x-ray one month postoperatively.

**Figure 6:**
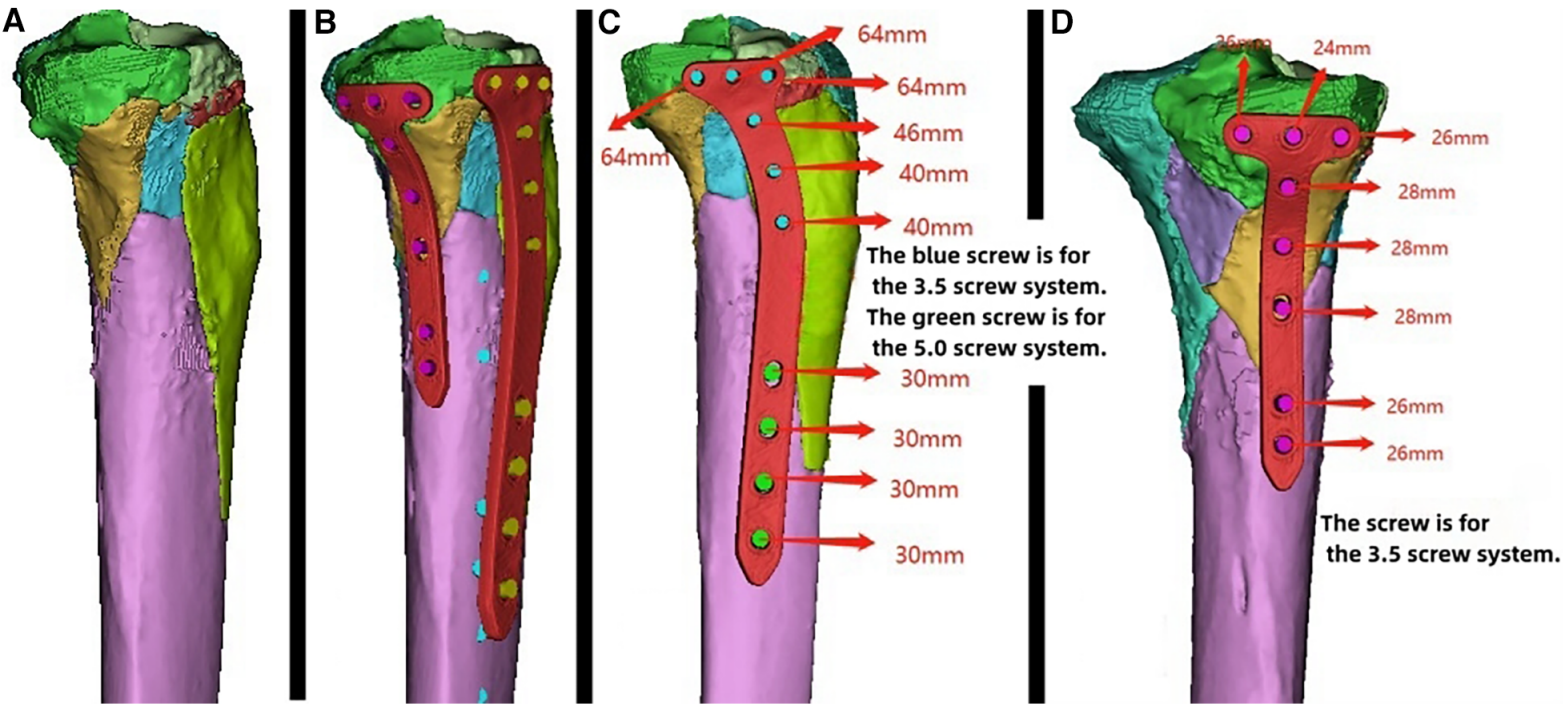
Patient's 3D printing and customized steel plate data. (**A**) Virtual simulation of preoperative CT data for fracture reduction. (**B–D**) Design of the steel plate and screws, simulation of the steel plate's position, and data for screw length measurement.

**Figure 7:**
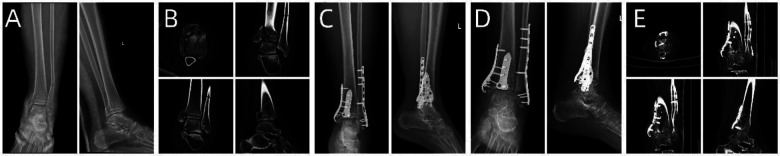
Patient's preoperative and postoperative radiological data. (**A,B**) Preoperative x-ray and CT images. (**C**) x-ray image on the first day after surgery. (**D,E**) x-ray and CT images 6 months after surgery.

**Figure 8:**
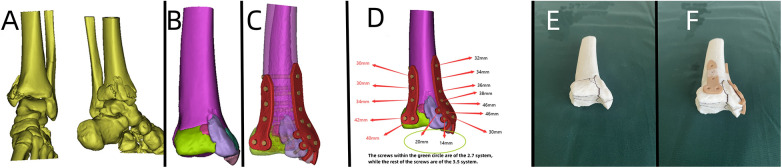
Patient's 3D printing and customized steel plate data. (**A**) Virtual simulation of preoperative CT data of the fracture. (**B**) Virtual reduction of the fracture data. (**C,D**) Design of the steel plate and screws, simulation of the steel plate's position, and data for screw length measurement. (**E,F**) 3D printing model and customized individualized steel plate model.

**Figure 9:**
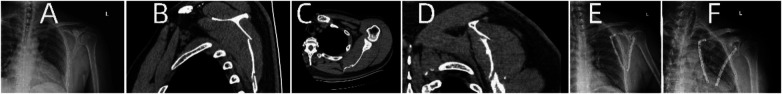
Patient's preoperative and postoperative radiological data. (**A**) Preoperative x-ray. (**B–D**) Preoperative CT. (**E**) x-ray on the first day after surgery. (**F**) x-ray one month after surgery.

**Figure 10:**
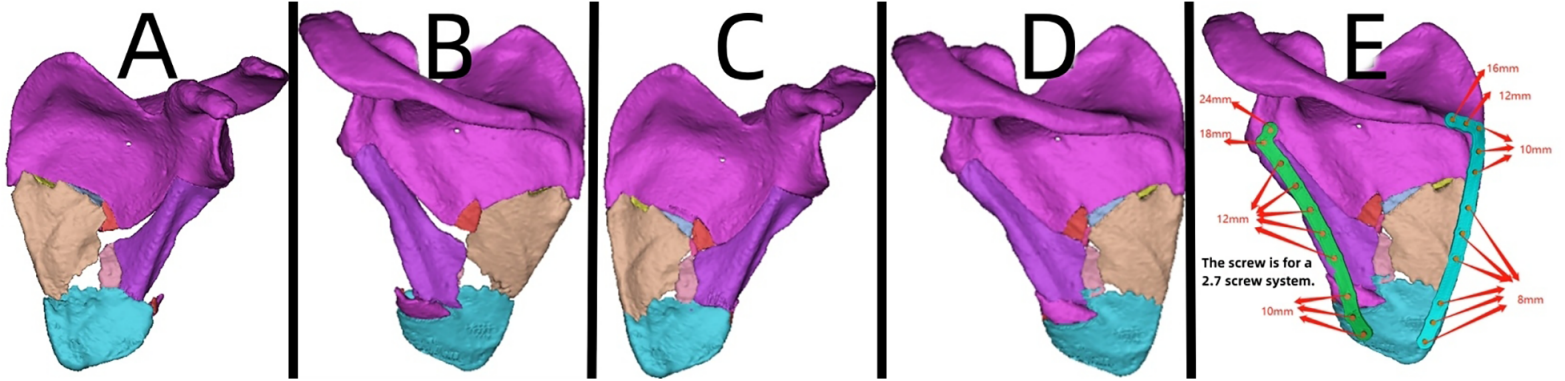
Patient's 3D printing and customized steel plate data. (**A,B**) Virtual simulation of preoperative CT data. (**C,D**) Virtual reduction of fracture data in the patient. (**E**) Design of the steel plate and screws, simulation of the steel plate's position, and data for screw length measurement.

Table 1 summarizes the cases in the 3D printing group. Table 2 compares the data of matched patients between the 3D group and the non-3D group.

**Table 1:** Individual case scenarios.

No.	A/B/G	Diagnosis	Fracture situation	DBS/NOS	OSO/FTP	Complications	Outcome (one year postoperative)
1	56/22.3/ Female	Fracture of the radial head and proximal Radius	Comminuted fracture, severe displacement, involving approximately 1/3 of the proximal radius	5/1	RHR or ORIF/ORIF	Yes/slight limitation of function	Satisfactory recovery of joint functions, including flexion, extension, and rotation, guided by the rehabilitation department.
2	49/29.4/ Male	Distal radius fracture	Highly comminuted fracture with significant displacement and collapse of the articular surface	4/1	WJR or ORIF/ORIF	No	Satisfactory recovery of wrist joint function, guided by the rehabilitation department.
3	43/25.6/ Male	Tibial plateau fracture/cranial trauma	Old, comminuted fracture with bony callus formation, bone defect, and collapse of the articular surface	26/3	ORIF/ORIF	No	Satisfactory recovery of joint function, with no limitations in flexion, extension, or walking.
4	33/28.7/ Male	Ankle joint fracture	Comminuted fracture with articular surface collapse, involving the inner, outer, and posterior aspects of the malleoli	3/1	ORIF/ORIF	No	Satisfactory recovery of joint function, with good ankle joint flexion, extension, inversion, and eversion.
5	44/31.2/ Male	Scapula fracture	Highly comminuted fracture with significant displacement	3/1	ORIF/ORIF	No	Satisfactory movement of the shoulder joint and scapula.

A/B/G, age/BMI/gender; DBS/NOS, days before surgery/number of surgeries; OSO/FTP, optional surgical options/final treatment plan; RHR, ORIF and WJR, radial head replacement surgery, Open reduction and internal fixation and Wrist joint replacement surgery.

**Table 2:** Comparison of related data between 3D group and Non-3D group patients.

	Fracture type	Age	Gender	BMI	Surgery time(min)	FRQ	JFS (*)	JFS (#)	ADL (#)	Healing time(weeks)	Complications
3D Group	Radial head	56	Female	22.3	73	Good	(Mayo) 95	95	95	11	Yes
	Distal radius	49	Male	24.2	62	Excellent	(Cooney) 95	95	100	12	No
	Tibial plateau	43	Male	21.6	99	Excellent	(HSS) 94	96	100	12	No
	Ankle joint	33	Male	23.7	90	Excellent	(Mazur) 93	96	100	11	No
	Scapula	44	Male	25.6	64	Excellent	(Neer) 95	97	100	12	No
Non-3D Group	Radial head	55	Female	23.1	95	Good	(Mayo) 85	90	95	13	Yes
	Distal radius	50	Female	23.8	86	Good	(Cooney) 85	91	95	14	Yes
	Tibial plateau	45	Male	22.3	118	Good	(HSS) 87	92	95	12	No
	Ankle joint	31	Male	23.4	120	Excellent	(Mazur) 86	89	95	13	No
	Scapula	42	Male	25.3	85	Good	(Neer) 88	91	95	13	No
P		0.944	1.000	0.911	0.059	0.206	<0.001	<0.001	0.032	0.016	0.524

FRQ, fracture reduction quality; JFS, joint function score; #, after one year post-operation; *, after 3 months post-operation.

The baseline characteristics were similar between the two groups. For the 3D group compared to the non-3D group, the age was (45.00 ± 8.46 vs. 44.60 ± 9.07, *P* > 0.05) and the BMI was (23.48 ± 1.58 vs. 23.58 ± 1.11, *P* > 0.05).

Comparison between the 3D group and the non-3D group revealed no significant difference in surgical time (77.60 ± 16.29 vs. 100.80 ± 17.08 min, *P* > 0.05), fracture reduction quality (excellent reduction rate 80% vs. 20%, *P* > 0.05), and complication rates (20% vs. 40%, *P* > 0.05). However, there was a significant difference in fracture healing time (11.60 ± 0.55 vs. 13.00 ± 0.71 weeks, *P* < 0.05), postoperative three-month joint function score (94.00 ± 1.00 vs. 86.20 ± 1.30, *P* < 0.05), postoperative one-year joint function score (95.80 ± 0.84 vs. 90.60 ± 1.14, *P* < 0.05), and postoperative one-year activities of daily living score (99.00 ± 2.24 vs. 99.00 ± 0.00, *P* < 0.05).

The combination of 3D printing technology and personalized customized steel plates provides patients with additional treatment options. Particularly in the treatment of complex fractures involving multiple joints, the use of 3D-printed customized implants has shown significant potential (Figures 1–8).

### 3.1 Case presentation

#### 3.1.1 Case 1

A 56-year-old patient with a radial fracture resulting from a car accident was assessed by CT (Figures 1A–D), revealing severe fragmentation and displacement of the radial head and proximal radius. Treatment options considered included open reduction internal fixation (ORIF) or radial head replacement. After discussing the options with the patient, the decision was made to proceed with ORIF. Virtual simulation based on the patient's data was performed (Figures 2A,B), followed by the creation of a 3D model and the design of a customized individualized steel plate (Figures 2C,D). This facilitated preoperative planning, simulation, and subsequent surgical intervention. The surgery was performed through a lateral incision on the elbow joint, with careful layer-by-layer dissection to protect the radial nerve. Upon exposure, the fracture was observed to be comminuted, with multiple fracture fragments. Reduction of the fracture was challenging during the procedure, but satisfactory realignment was achieved with reference to preoperative fracture models and disinfection. Temporary fixation was achieved using Kirschner wires, and intraoperative fluoroscopy confirmed satisfactory fracture reduction. The fixation employs individualized steel plates with customized shapes and hole positions provided by Shenyang Dongya Company. Post-fixation, the elbow joint showed stable fracture alignment, which was confirmed by intraoperative fluoroscopy using a C-arm. The site was irrigated, and the incision was sutured. The site was irrigated, and the incision was sutured. Postoperative imaging (Figures 1E–H) and the final follow-up follow-up assessments demonstrated the patient's successful recovery of joint functionality, with satisfactory flexion, extension, and rotation capabilities.

#### 3.1.2 Case 2

A 49-year-old patient with a distal radial fracture resulting from a fall was examined by CT (Figure 3), revealing a highly fragmented fracture with displaced bone fragments and severe joint surface collapse. Considering the patient's relatively young age, wrist joint replacement and open reduction internal fixation (ORIF) were initially considered. However, the patient and family opted for the latter. Due to post-injury soft tissue swelling, the patient was managed for swelling reduction, and surgery was deferred until the soft tissue condition improved. Virtual simulation based on the patient's data was performed (Figures 4A–D), followed by the creation of a 3D model and the design of a customized individualized steel plate (Figures 4E,F). This facilitated preoperative planning, simulation, and subsequent surgical intervention. The surgery was performed using two incisions, one on the volar aspect and the other on the dorsal aspect, with layer-by-layer dissection while carefully protecting the median nerve. The fracture was exposed, revealing comminution and displacement of fracture fragments. Despite difficulty in reducing the fracture during the procedure, satisfactory realignment was achieved by referencing preoperative fracture models and disinfection. Temporary fixation was achieved using Kirschner wires, and intraoperative fluoroscopy using a C-arm confirmed satisfactory fracture reduction. The fixation employs individualized steel plates with customized shapes and hole positions provided by Shenyang Dongya Company. Post-fixation, the wrist joint exhibited stable fracture alignment, which was further confirmed by intraoperative fluoroscopy using a C-arm. The site was irrigated, and the incision was sutured. The final follow-up follow-up assessments demonstrated the patient's successful postoperative functional recovery.

#### 3.1.3 Case 3

A 43-year-old patient involved in a car accident with traumatic brain injury, tibial plateau fracture, and an open wound on the left lower limb underwent hospitalization for wound debridement and treatment of the head injury. x-rays and CT scans (Figures 5A–D) revealed a comminuted fracture of the tibial plateau. Once the patient's overall condition stabilized, it was observed that the tibial fracture had become chronic. A 3D model was printed to assess the immediate status of the fracture, plan the surgery, and evaluate bone callus and defect conditions. Based on the patient's relevant data, a virtual simulation was conducted (Figure 6A), and a customized individualized steel plate was designed (Figures 6B–D) for preoperative planning, simulation, and subsequent surgical intervention. The surgery was performed through a medial curved incision on the tibia, with step-by-step dissection of the skin and subcutaneous tissues to expose the fracture. The fracture was observed to be comminuted, with collapse of the articular surface and partial formation of callus. Despite difficulty in realigning the articular surface intraoperatively, satisfactory reduction of the fracture and articular surface was achieved by referencing preoperative fracture models and disinfection. Temporary fixation was accomplished using Kirschner wires, and intraoperative fluoroscopy with a C-arm confirmed satisfactory realignment of the fracture and articular surface. The fixation employs individualized steel plates with customized shapes and hole positions provided by Shenyang Dongya Company. Post-fixation, the knee joint exhibited stable alignment of the fracture, which was further confirmed by intraoperative fluoroscopy with a C-arm. The site was irrigated and the incision was sutured. The final follow-up follow-up assessments, including imaging studies (Figures 5E,F), and functional follow-ups, demonstrated that the patient achieved a favorable recovery in joint function, with satisfactory outcomes in flexion, extension, and ambulation.

#### 3.1.4 Case 4

A 33-year-old patient involved in a car accident sustained a trimalleolar ankle fracture. Given the patient's young age, achieving the best surgical outcome and satisfactory postoperative functional recovery were the primary goals. Preoperative x-rays and CT scans (Figures 7A,B) revealed severe comminution of the fracture, involving the medial malleolus, lateral malleolus, and posterior malleolus, with joint surface collapse and displacement of fracture fragments.

Based on the patient's relevant data, a virtual simulation was conducted (Figures 8A,B), and a customized individualized steel plate was designed (Figures 8C,D). 3D models of the fracture and the customized plate were printed (Figures 8E,F) for preoperative planning, simulation, and subsequent surgical intervention. The surgery was performed through two incisions, one on the medial and the other on the lateral aspect of the distal tibia and fibula, with step-by-step dissection of the skin and subcutaneous tissues to expose the fracture. The fracture was observed to be comminuted, with displacement of the posterior malleolar fragment. Reduction of the fracture was challenging intraoperatively; however, satisfactory realignment was achieved by referencing preoperative fracture models and disinfection. Temporary fixation was achieved using Kirschner wires, and intraoperative fluoroscopy with a C-arm confirmed satisfactory fracture reduction. The fixation employs individualized steel plates with customized shapes and hole positions provided by Shenyang Dongya Company. Post-fixation, stable alignment of the ankle joint was observed, which was confirmed by intraoperative fluoroscopy with a C-arm. The site was irrigated, and the incisions were sutured. The final follow-up assessments, including imaging studies (Figures 7C–E), and functional follow-ups demonstrated that the patient achieved a favorable recovery in joint function after surgery.

#### 3.1.5 Case 5

A 44-year-old patient suffered a shoulder blade fracture due to a fall, and the patient was obese. Preoperative x-rays and CT scans (Figures 9A–D) revealed a severe and complex fracture. Based on the patient's relevant data, virtual simulations were performed (Figures 10A–D), and 3D models of the fracture were printed. A customized individualized steel plate was designed (Figure 10E) for preoperative planning, simulation, and subsequent surgical intervention. The surgery was performed through two incisions, one on the inner and the other on the outer edge of the scapula, with step-by-step dissection of the skin and subcutaneous tissues to expose the fracture. The fracture was observed to be comminuted, with displacement of fracture fragments. Reduction of the fracture was challenging intraoperatively; however, satisfactory realignment was achieved by referencing preoperative fracture models and disinfection. Temporary fixation was achieved using Kirschner wires, and intraoperative fluoroscopy with a C-arm confirmed satisfactory fracture reduction. The fixation employs individualized steel plates with customized shapes and hole positions provided by Shenyang Dongya Company. Post-fixation, stability of the fracture was observed with movement in various directions of the shoulder joint, which was further confirmed by intraoperative fluoroscopy with a C-arm. The site was irrigated, and the incisions were sutured. The final follow-up follow-up assessments, including imaging studies (Figures 9E,F), and functional follow-ups demonstrated that the patient achieved satisfactory surgical outcomes and postoperative functional recovery.

## 4 Discussion

With the advancement of orthopedics, surgery is no longer limited to merely relieving patient pain, fixing fractures, and correcting deformities. It now aims to not only address these issues but also enhance the restoration of patients' limb function. Limbs, as the primary sites for motor function, demand greater precision in fracture reduction and higher postoperative functional recovery. The use of 3D printing technology in combination with personalised custom-made plates allows for the simulation of surgical reduction and procedures based on models and individualized plates. During surgery, it enables comprehensive fracture reduction and provides more robust and stable internal fixation. This technology facilitates the measurement of screw lengths and the assessment of incision locations and lengths, ensuring a smooth and secure surgical procedure (9). Bagaria and Chaudhary (43). conducted a multicenter study, which demonstrated the crucial role of 3D models in complex orthopedic surgeries, such as complex fractures and fractures around joints. This technology not only provides additional information beyond traditional imaging but also offers significant support in preoperative planning, surgical rehearsal, procedural simulation, intraoperative guidance, surgical navigation, pre-implant selection, and inventory management. It holds the potential to reduce surgical time and enhance surgical precision, garnering strong support and recommendations from surgeons. The research by Upex et al. (44) indicates that utilizing 3D printing technology for preoperative planning significantly enhances the quality of fracture reduction and reduces surgical time. You et al. demonstrate that models generated using 3D printing technology can provide a visual representation of the severity of fractures and enable comprehensive observation. This aids in preoperative diagnosis, surgical planning and design, data measurements, preselection of internal fixation devices, and surgical outcome simulation. It assists surgeons in gaining a better understanding of complex fracture cases, designing the most suitable surgical approach before the operation, facilitating doctor-patient communication, reducing intraoperative damage, and optimizing surgical outcomes (45). Xie et al.'s research indicates that for tibial plateau fracture patients, 3D printing technology assisted ORIF is a more suitable method for treating tibial plateau fractures. It results in shorter surgical duration, reduced intraoperative bleeding, and faster healing time (46).

Giannopoulos et al.'s study (47) demonstrates that 3D printing models can enhance diagnosis, facilitate advanced preoperative planning, and enable surgeons to absorb information more rapidly than image reviews alone. These models assist in selecting the optimal surgical approach. Patient-specific 3D printed implants may have a significant impact on future surgeries. This research also indicates that 3D printing technology is rapidly evolving and could potentially be a transformative factor for surgeons. However, Rengier et al. (21) argue that while 3D printing and rapid prototyping are feasible for specialized surgical planning and prosthetic applications, their widespread use in preoperative surgical planning and personalised implant design seems impractical. This is because standard planning procedures and standardized implants are already sufficient, and the time required for creating 3D printed models is relatively long, making it unsuitable for emergency patients. However, the authors have found through clinical practice that complex limb fractures often involve severe fragmentation and significant displacement. Using 3D printing technology to create limb fracture models allows physicians to gain a clear understanding of the fracture location and type. Customising individualized plates through these models enables more comprehensive and robust fixation of fractures while also saving surgical time. Currently, 3D printing technology can provide a relatively comprehensive surgical approach and planning for complex limb fractures. However, whether it significantly reduces surgical time, decreases the probability of soft tissue damage, improves fracture reduction quality, and enhances postoperative function for all fracture surgeries remains to be confirmed and further studied, depending on the specific fracture case.

With the continuous research and advancements in using biomaterials in 3D printing, the applications of 3D printing in manufacturing customized implants, prosthetics, and medical 3D scaffolds have been rapidly growing (33). Customized internal implants make each patient unique, holding tremendous potential in individualized and tailored solutions (32). Today, 3D printing technology can be used to create physical models, plan surgeries, or develop surgical guides, aiding orthopedic surgeons in dealing with complex cases. Building upon this foundation, Mimics computational software is employed to create perfectly tailored individual internal fixations based on different fracture locations and types, eliminating the need for preoperative simulation of pre-bent plates. This further realizes the customisation of surgical treatments and has achieved satisfactory clinical outcomes. The use of 3D printing technology and customisation in orthopedics has become a reality (4). Yoshii et al. (48) employed three-dimensional preoperative planning for the treatment of distal radius fractures. The reproducibility of fracture reduction was relatively high, and the reproducibility of implant selection was outstanding. Three-dimensional digital planning is highly useful for visualizing the reduction process and choosing appropriate implants to address distal radius fractures. Furthermore, Yoshii et al. (49) demonstrated the significant benefits of preoperatively determining the plate and screw positions and types for distal radius fractures through 3D printing and image fusion systems. Shuang et al. (50) compared the treatment of intercondylar fractures of the humerus using traditional fixed plates and 3D printing technology with customized individualized plates, with the latter achieving more satisfactory postoperative joint function recovery. This demonstrates that the use of 3D printing technology in conjunction with personalised custom plates is safe and effective for treating intercondylar fractures of the humerus and significantly reduces the surgical time. The study by Yang et al. (51) demonstrates that 3D printed custom patient-specific plates are effective in surgical procedures, simplifying the surgical process and achieving precise surgical outcomes through highly accurate methods.

In recent years, the author has successfully utilized 3D printing technology in conjunction with personalized custom steel plates to treat a series of complex limb fractures. For patients with such intricate fractures, it is crucial to recommend more and better treatment options. Additionally, for some younger patients, there is a strong desire to preserve their own bone structure and joints rather than opting for prosthetics or internal implants. For physicians, the pursuit of minimal trauma, shorter surgery times, perfect, comprehensive, and robust fracture fixation to promote rehabilitation and achieve better joint functional recovery is a priority. These principles motivated the author to undertake the use of 3D printing technology combined with personalized custom steel plates for the treatment of complex limb fractures.

Reviewing literature and practical surgical experiences revealed that collaborating with engineers to design customized individualized steel plates using 3D printing technology resulted in a perfect fit during surgery. This approach successfully achieved fracture reduction and comprehensive, robust, and stable fixation, reducing surgical complexity. It also provided conditions for early initiation of functional exercises, positively impacting patient prognosis.

However, challenges persist with 3D printing technology combined with personalized custom steel plates, including extended customization times, high costs, and exclusion from medical insurance settlements (36). The author believes that with the advancement of technology, these issues will gradually be overcome, offering patients higher quality and more satisfactory services.

The limitations of this study include a small sample size of included cases, potential selection bias, and a lack of long-term follow-up of 3 to 5 years. These factors may introduce some bias into the results. Additionally, the widespread application of this surgical method is limited due to the incomplete popularization of 3D printing technology. This study is a case series with limited persuasiveness and credibility, and the data reporting and comparisons are not comprehensive. It is hoped that in the future, more high-quality randomized controlled studies with larger and more diverse samples will be conducted to provide more comprehensive and accurate conclusions.

## 5 Conclusion

The combination of 3D printing technology with personalized custom steel plates has created more choices and convenience for preoperative planning, treatment option selection, and surgical procedures for patients with limb fractures. This approach provides a more precise and personalized therapeutic method, realizing personalized medicine, aiding in reducing surgery time, minimizing trauma, and improving patient functional recovery. This technology is poised to become the trend in the surgical treatment of various complex traumatic bone fractures.

## Data Availability

The raw data supporting the conclusions of this article will be made available by the authors, without undue reservation.
